# Exploring parental opinions on oral hygiene behavior and knowledge of their young children in Lithuania: a cross-sectional survey study

**DOI:** 10.3389/froh.2025.1530265

**Published:** 2025-04-29

**Authors:** Yvonne A. B. Buunk-Werkhoven, Rasa Tamulienė, Daiva Mačiulienė

**Affiliations:** Faculty of Medicine, Oral Health Department, Kauno Kolegija Higher Education Institution, Kaunas, Lithuania

**Keywords:** oral health promotion, oral hygiene behavior, oral health knowledge, parents, pre- and primary school children, Lithuania, behavioral sciences, public health

## Abstract

**Background:**

An appropriately formulated oral health education program carefully based on research, can increase knowledge, change behavior in a positive direction and improve self-confidence. This study aimed to examine parental opinions on their children's oral hygiene behavior (OHB) and oral health knowledge (OHK) among their pre- and primary school children in Kaunas, Lithuania.

**Methods:**

In this cross-sectional study, an online 33-question survey was conducted before and after World Oral Health Day on March 20 to assess the oral hygiene skills, eating habits, and demographics of their 5–12 year children. A total of 532 parents participated, with data from 420 parents, mainly married mothers (average age 37.3 years) being analyzed. Most participants had higher education, lived in Kaunas, and had one to three children, with an average age of 7 years for the oldest child.

**Results:**

Most participants used a manual toothbrush. The adapted OHB index showed that most parents generally had good control over their children's tooth brushing habits, with many brushing twice daily and using fluoride toothpaste. One-third of parents always re-brushed their child's teeth after the child brushed independently. Parents demonstrated strong knowledge of their children's oral health care, as reflected in high scores on the adapted OHK index. A positive correlation was found between OHB and OHK (*r* = 0.14, *p* = 0.05). Younger children were re-brushed more frequently, and higher parental OHK was linked to more frequent re-brushing, particularly for children less than 10 years, and parents with higher education had better OHK but did not demonstrate better OHB.

**Conclusions:**

The insights gained from parents into their children's OHB and OHK can help implement an evidence-based preventive approach to improve their children's oral hygiene practices.

## Introduction

The Lancet Commission on Commercial Determinants of Health highlights unhealthy diets as a key contributor to disease burden and mortality, significantly impacting oral health inequalities, especially concerning sugars and overall health (1). The World Health Organization (WHO) states that tooth decay is one of the most prevalent childhood diseases and is widespread in many countries. In Lithuania, public health in dentistry must equally meet the objectives of the WHO Global Oral Health Action Plan 2020–2030 (2) to improve health outcomes in the community. Despite active preventive programs, tooth decay rates among children remain high (3). Data from Lithuanian Public Health Offices and the Ministry of Health (4) indicate that, over the past six years, the percentage of children with healthy teeth has only increased by 4.3%. The highest percentage of children with healthy teeth is seen in preschoolers, with about 60% maintaining good oral health. In contrast, only 22.7% of children aged 7–17 have healthy teeth, indicating nearly three times fewer healthy cases among those in educational settings. Data from the Children’s Health Monitoring Information System show (4) a significant decline in oral health once children start school, with only around 17% of 7- to 8-year-olds having healthy teeth. Maintaining optimal oral hygiene and nutritional habits is essential for preventing oral diseases, such as dental caries, in children. The ages of 6–12 years are particularly vulnerable as children lose primary teeth and develop permanent ones. During this period, children gain independence in their oral self-care routines and their dietary choices, making it crucial to guide parents on appropriate oral hygiene behavior, eating habits, and factors influencing caries and occlusion development (5, 6). Therefore, it is essential for children in this age group to learn these habits early on with support from especially mothers and other caregivers (7, 8). In Lithuania, current preventive measures for oral diseases in children include mandatory annual oral health check-ups and a molar sealing program, performed by dentists with dentists or dental hygienist (9), together with occasional lessons by public health or oral care specialists in schools. However, these preventive public health measures appear effective only in the short term, lack proper implementation within a supportive system, and frequently do not engage parents. Nevertheless, recent studies emphasizes that implementing various programs in schools to promote oral hygiene habits effectively enhances oral health, prevents diseases, and to improves children’s oral hygiene habits (10, 11). These school-based preventive programs have been shown to positively influence children's behaviors related to oral health, particularly through environmental modifications (5) that teach effective tooth brushing techniques and encourage comprehensive oral hygiene at home. Tooth brushing plays a crucial role for effective plaque control and adequate oral hygiene behavior (OHB) relies not only on oral health knowledge (OHK) and attitude towards this habit behavior, but also on the effectiveness of the specific method and the ease with which parents and children can perform the procedure (12–15).

### Overview of present research

This study aimed, using the recently modified indices of oral hygiene-related behavior (OHB) and oral health knowledge (OHK) for parents of pre- and primary school children (16, 17), to assess OHB, OHK for the developing of an oral hygiene intervention, i.e., a Smart Application, to promote oral hygiene habits in children aged 5–12 years. A reasonable assumption is that a positive correlation between parents' OHK and the level of OHB means that parents with better oral health knowledge are more likely to implement positive oral hygiene practices and influence their children's oral health behaviors.

## Materials and methods

### Study design and setting

This descriptive cross-sectional human study, conducted in parents of pre-primary and primary school children (16, 17), was administered to a sample of the Lithuanian population from 13 to 28 March 2024. The survey including the two adapted indices OHB and OHK was uploaded to the Kauno Kolegija survey system, LimeSurvey. The distribution and access of the survey link (18) was strategically planned around World Oral Health Day on March 20 to increase interest and participation, as schools often organize various health-related events during this period. A representative of the Public Health Office sent an invitation to participate in the survey to public health specialists working in Kaunas schools implementing preschool and primary education programs. These specialists shared the invitation, including a survey link, via internal communication channels (e.g., e-mail, electronic diary message systems) used in the schools with all parents of children aged 5–12 years.

### Participants

The included participants were parents of preschool and primary school children in schools in the city of Kaunas. For this study, permission was obtained and approved by the Applied Scientific Research Ethics Compliance Committee of Kaunas Kolegija HEI (No.13-14). The study was conducted according to universal ethical principles, in line with the Helsinki declaration, and in collaboration with the Kaunas Public Health Bureau in Lithuania. Participation was voluntary, no personally identifiable information was collected to ensure confidentiality, and anonymity was guaranteed.

### Study size

For the sample size, non-probability sampling method was used, and it was calculated that a sample of 374 parents would guarantee a reliable study with a confidence interval of 95%. The questionnaire was viewed and opened by five hundred and thirty-two participants. Of this amount of 532 respondents, 11 refused to participate after reviewing the purpose and content of the study, which was described in the introductory section. 521 respondents completed the online questionnaire, and after checking and exploring the data, 20 responses were excluded due to incorrect completion (e.g., specifying the age of several children instead of one, specifying the age of the child instead of the age of the parent, etc.). Therefore, 501 responses were considered suitable for further analysis. In addition, 81 respondents were excluded because they did not meet the criteria (they had children younger than five or older than 12).

#### Questionnaire

The questionnaire development followed a two-round Delphi method (19, 20) and was described in detail in Lithuanian (17). This Lithuanian version, including the adapted OHB and OHK indices, were translated into English using the forward-backward procedure (21). The introductory section of the questionnaire outlined the study's purpose, how results would be utilized, and provided contact information. Participation was voluntary, with respondents choosing to agree or decline. Those who declined were directed to the end of the questionnaire. No personally identifiable information was collected, ensuring confidentiality. All data were used only in aggregate form, so that confidentiality was assured. The 33-item questionnaire included demographic questions (gender, age, education, place of residence, family status, number of children in the family, and the child's gender and age) and assessed parents’ knowledge, behaviors regarding children's oral health, and motivational methods using multiple-choice, bipolar adjective ratings, or Likert scales.

### Bias

The study used a non-probability sampling method, meaning participants were not randomly selected. Responding parents may have had a greater interest in health-related topics, leading to selfselection. Furthermore, the survey was distributed through school communication channels, which may have excluded parents who are not actively involved in these platforms. Since the questionnaire was self-administered and anonymous, there is a risk of social desirability bias, where respondents may have provided answers that they considered more socially acceptable rather than their actual behavior or opinions. Some of the answers (*N* = 101) were excluded due to incorrect completion or not meeting the inclusion criteria. This could have introduced bias if the excluded participants were systematically different from those included in the final analysis. Some survey questions may have required parents to recall past behaviors or events, raising the possibility of inaccurate reporting due to memory limitations. The survey was conducted around World Oral Health Day, a time when health awareness may have been higher. This could have temporarily influenced parents’ attitudes or behavior, potentially affecting the generalizability of the findings to other time periods.

### Oral hygiene behavicor

This careful detailed behavior was measured using the adapted index for OHB for pre-school and primary school children (16, 17), which maintained all original criteria (12), except for interdental care. Parents were explicitly asked to indicate what type of toothbrush their children used: a manual toothbrush, a powered toothbrush, or a combination of both types of toothbrushes. The adapted index consists of 7 items, e.g., the item ‘My child brushes her/his teeth:’ was supported by pictures showing different toothbrushing methods, and there were some differences in item weight assignments. For example, the expert group decided to retain the item on brushing force, acknowledging the subjectivity of parental assessments while recognizing its value in understanding perceived optimal pressure, and they recommended brushing teeth in the morning before breakfast and in the evening, aligning with new FDI guidelines (22) (see Table 1). After the item values were calculated, a computed sum OHB score could range from 0 to 16. The higher the sum score, the more optimal the described oral hygiene-related behavior is to ensure good self-care oral hygiene behavior. And logically, the higher the child's total score, the more suitable the child's oral hygiene-related behavior will be to maintain healthy teeth and thus ensure the prevention of oral diseases. Since the original OHB index (12) is intended to indicate actual or reported oral hygiene behavior, the OHB index is not a scale and calculating reliability (Cronbach's α) to check the internal consistency of the items is not very meaningful and therefore unnecessary (23).

**Table 1:** Oral hygiene-related behavior Index for pre-school and primary school children (16, 17); a total score (*N* = 420), and for those who reported to use a manual toothbrush (*N* = 240); percentages for each item.

Items	Values	Total	Manual toothbrush
Frequency of toothbrushing	‘Not every day’ ‘Once a day’ ‘Twice a day’ or ‘More than 2 times a day’	4.5 33.3 62.2	5 35 60
The time of day when the teeth are brushed (17)	‘Once a day’ or ‘Twice a day’ (weight: 1) ‘Twice a day’ (weight: 2) ‘Twice a day’ (weight: 3) ‘Three or more times’ (weight: 4)	31.6 32.5 26.6 9	34.6 31.3 24.2 10
Pressure forces when brushing teeth	Softly (‘1, 2, 3’) Softly/Forcefully (‘4, 5’) With strength (‘6, 7’)	47.1 51 1.9	49.6 49.2 1.3
The duration of toothbrushing in minutes	‘Two minutes’ or ‘Three minutes’ ‘Longer than three minutes’ or ‘One minute’ Less than ‘One minute’	55.2 36.4 8.3	51.3 39.6 9.2
Toothbrushing movements	‘Bass method’ ‘horizontal method’(‘forward-backward’ movement) ‘vertical method’ (up-down movement) and ‘circular method’ (movement in a circle)	6.4 32.6 61	3.7 36.3 59.9
Type of toothpaste	‘Toothpaste with fluoride’	79.5 20,5	79.2 20.8

### Oral health knowledge

The 16 items in the modified OHK index (16, 17) reveal the status of the knowledge of parents about the oral health of their children, and covered three areas: parents’ knowledge about children's individual oral hygiene at home (items 2, 3, 4, 5, 6, 9), parents’ knowledge about the influence of nutrition on children's oral health (items 11, 12, 14, 15) and parents’ general knowledge about the factors determining children's oral health (items 1, 7, 8, 10, 13, 16) (Table 2). Some examples are, ‘To keep the child's teeth healthy, it is important that the toothpaste contains fluoride’, ‘The sugary drinks that the child consumes can affect the condition of the child's teeth’, Solid food helps to form a correct bite’, ‘It is more important to take care of the condition of children's permanent teeth, rather than their milk teeth’, and ‘Children under the age of 10 cannot brush their teeth optimally, so their parents must (re)brush them afterwards’. For all statements parents had to choose one option: 1 = *true* or 0 = *false*. With the exception of item 7, all other items were true. A sum score was computed, so that a total OHK score was formed for each respondent (ranging from 0 to 16). The higher the total score, the higher the better the knowledge of parents about the oral health of children. Due to the limited number of dichotomous items, the OHK index was not deemed a valid scale. However, its face validity for parents of preschool and primary school children was considered acceptable.

**Table 2:** Oral health knowledge Index for pre-school and primary school children (16, 17); a total score (*N* = 420). Percentages correct answers for each statement.

Items	Statements about the oral health care of children	Total
1	A child should visit an oral health care professional/specialist at least once a year	98.1
2	To keep the child's teeth healthy, it is important that the toothpaste contains fluoride	68.3
3	The child should brush his/her teeth twice a day	98.1
4	Brushing your teeth regularly with toothpaste and brushing your teeth twice a day can help prevent tooth decay	96.2
5	It is important to ensure that the child cleans all surfaces of the teeth	98.6
6	The amount of toothpaste placed on the toothbrush should not be larger than a pea size	91.2
7	It is more important to take care of the condition of children's permanent teeth, rather than their milk teeth	87.1
8	Sealing the teeth (covering the fissures of the molars with a special material) helps to ensure that the child's teeth are healthy	82.6
9	A child's toothbrush should be changed every three months	91.2
10	Parents should choose oral health care products for their child based on the recommendations of oral health care professionals/specialists	96.9
11	The sugary drinks that the child consumes can affect the condition of the child's teeth	98.8
12	Sweets should be avoided to keep the child's teeth healthy	91
13	It is important to ensure that the child does not have any more snacks or drinks after brushing her/his teeth in the evening	99.3
14	Nutrition affects a child's oral health	99
15	Solid food helps to form a correct bite	78.1
16	Children under the age of 10 cannot brush their teeth optimally, so their parents must (re)brush them afterwards	75.5

### Statistical analysis

The collected data were downloaded into Excel and in IBM Statistical Package for Social Sciences 29.0 (SPSS Inc., Chicago, IL, USA) files for further data analysis. The calculations included descriptive statistics; the data were subjected to frequency distributions, and means and standard deviations (SDs). Interscale correlation on OHB and OHK was assessed using Pearson's correlations. Also, chi-square and ANOVA tests were used for group comparisons (parental education level, children ages), and calculation of means for quantitative measures. Differences were considered statistically significant at *p* < 0.05.

## Results

In total 420 parents with a mean age of 37.3 years (SD = 4.6, ranging 24–55) responded to the survey. 395 (94%) of the parents were female and one respondent didn't want to reveal. Almost all of the parents come from the city, only 10.7% (*N* = 45) lives in the Kaunas district; 355 (84.5%) mainly mothers had higher education, 14% of the sample (*N* = 59) had a secondary or vocational level of education, and only 6 respondents reported an elementary level of education. 82.6% of the parents (*N* = 347) were married, 37 (8.8%) respondents were living with a partner, and the rest were not married, divorced or widowed. The number of children in the 420 family situations varies from an average of 1.8 (SD = 0.7), 49% boys, 50% girls, and 4 without revealing. Just over half of the parents (55.2%) have two children, about a third (31.9%) has one child, and 11.2% reported 3 children. In the remaining 1.6%, 6 respondents have 4 children and 1 person has 5 children. The mean age of the oldest child of the parents in this sample was 7.35 years (SD = 1.9, ranging 5–12).

### Oral hygiene behavior

**Figure 1:**
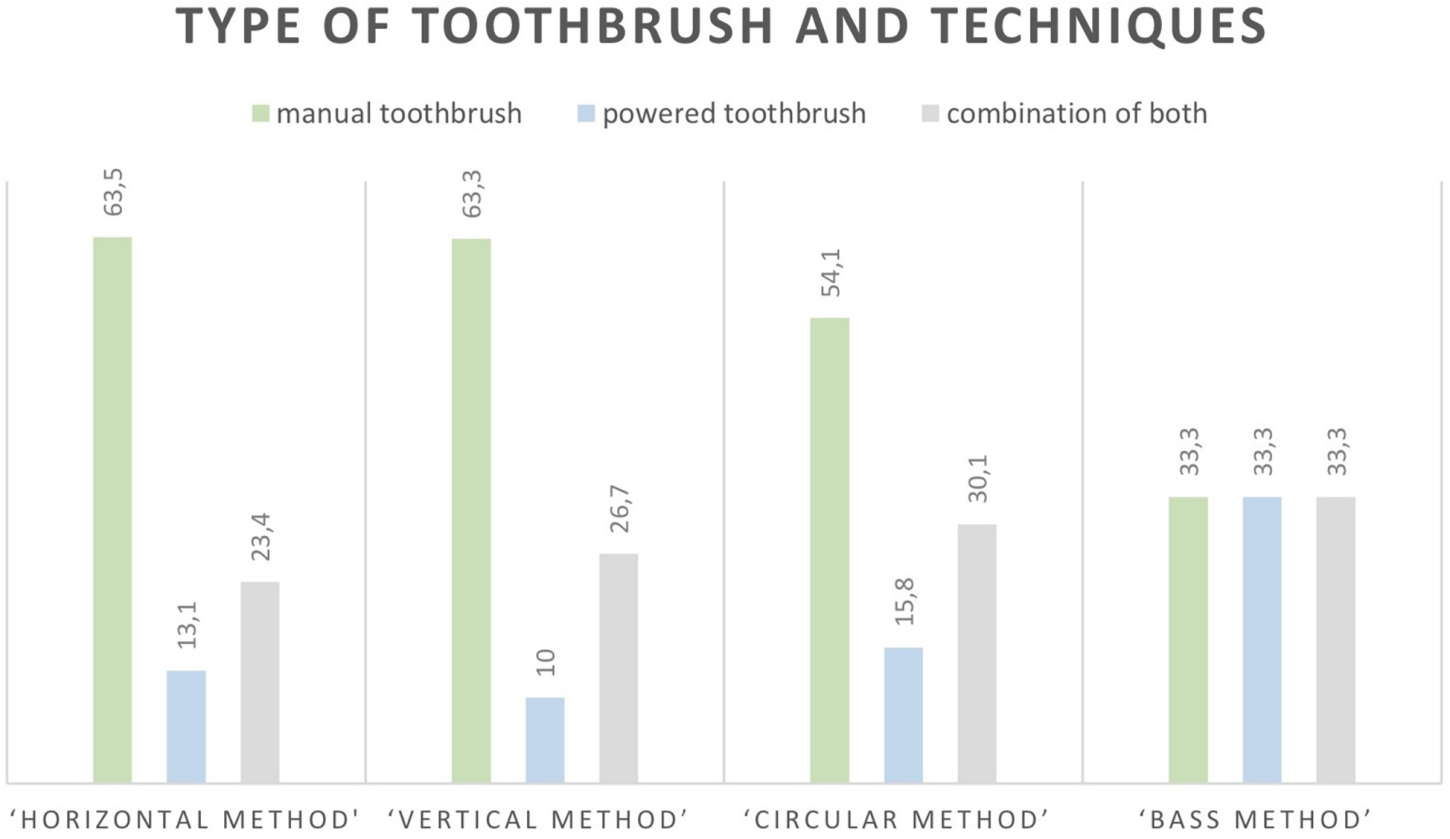
Percentages in tooth brushing techniques (*N* = 420); “horizontal method” (*N* = 137); “vertical method” (*N* = 60); “circular method” (*N* = 196); “bass method” (*N* = 27).

In the total sample, more than a half (57.1%, *N* = 240) reported to use a manual toothbrush, 64 respondents reported to use a powered toothbrush (15.2%), and more than a quarter (27.6%; *N* = 116) reported a combined use of both types of toothbrushes. The frequencies in percentages of the items concerning the adapted OHB index from parent's perspective towards their children, for the total sample and for those who reported to use a *manual* toothbrush (*n* = 240) are presented in Table 1. The mean, standard deviation and range of the total score on the adapted OHB index was for the total sample *N* = 420, M = 8.6 (SD = 2.0, ranging 3–14), the distribution of scores was approximately normal. For participants who reported using a manual toothbrush, the mean and standard deviation were the same, but the maximum score in this subgroup ranged up to 13. The total OHB score indicated children's oral hygiene care practices from parent's perspective, and here, the parents had adequate control over the oral hygiene care practices of their children. For instance, the findings with the OHB index showed that three fifths of the parents reported that their children brushed their teeth twice a day, and a little over a quarter (26.6%) brush their teeth in the morning before breakfast and in the evening before bedtime as recommended by the Lithuanian expert group (16). Only 9% ensured optimal oral hygiene by brushing their teeth three or more times a day. Almost 80% used fluoride containing toothpaste, as recommended by professionals worldwide (22). Less than 10% cleaned their tongue daily and one third (30%) never did so. In this sample, differences in toothbrushing techniques were found between those who used a manual toothbrush, a powered type of toothbrush or a combination of both types, *X*^2^ (6, *n* = 420) = 13.37, *p* = .038). As Figure 1 shows the ‘horizontal’ method (i.e., short scrubbing or in a ‘forwardbackward’ movement), the ‘vertical method’ (up-down movement), and the ‘circular method’ [i.e., gentle circular motions, so called ‘Fones technique’ (24, 25)] were used most by those who used a manual toothbrush, next by those who used a combination of both, and least by those who used a powered type of toothbrush. Although the ‘Bass method’ is considered by the professional group to be the most suitable brushing movement when using a *manual* toothbrush (12, 13, 17), only 3.7% of the group that brushes their teeth with a manual toothbrush indicated that they used this brushing technique (Table 1). It did not matter whether manual, powered or alternating between both types of toothbrushes were used, in all cases this was 33.3% (Figure 1). These findings do not directly show what those who combine both types of toothbrushes which toothbrushing techniques they use for the manual and powered toothbrush. No differences in toothbrushing details were found between those who used a manual, a powered or a combination of type of toothbrush. Only for the duration of toothbrushing there was a marginal significant difference, *X*^2^ (4, *n* = 420) = 8.8, *p* = .067. For the specific question about ‘re-brushing by parents’, approximately one third of parents (31.6%) indicated that they always re-brushed their child's teeth after the child had brushed independently, less than half of parents (46.3%) sometimes re-brushed their child's teeth, and one in five (21.9%) indicated that they never re-brushed their child's teeth. No differences were found in parents’ rebrushing their child's teeth after the child had brushed independently between those who used a manual, a powered or a combination of toothbrush types.

### Oral health knowledge

The frequencies in percentages of the items concerning the OHK for parents raising children of preschool and primary school age are presented in Table 2. The mean score with standard deviation and the range value of the OHK was M = 13.8 (SD = 1.4, ranging 3–16). It can be seen that parents’ knowledge of their children’ oral health care was very good. About one-third (33.3%; *N* = 140 and 29.3%; *N* = 123) of the parents had a sum score OHK of 14 and 15, respectively, and even 19 parents (4.5%) had all the answers correct. Statements 2, 7, 8, 15, and 16 were answered correctly by a lower percentage of parents, ranging from 68% to 87%. No significant differences were observed between the groups using manual, powered, or a combination of both types of toothbrushes.

In the total sample, there was a significant difference between child's tooth re-brushing by parents for the different age groups, *X*^2^ (4, *n* = 420) = 80.0, *p* = .000. The younger the children, the more often parents re-brush their children's teeth. This did not matter what the parents’ level of education was. However, there is a consistency between parental oral health knowledge and parental oral hygiene behavior; the more parents know that children under 10 years need to have their teeth rebrushed, the more often parents actually re-brush their children's teeth, *X*^2^ (4, *n* = 420) = 74.35, *p* = .000.

In the subgroup who used a manual toothbrush (*N* = 240), parents’ perspectives on theirchildren's oral hygiene behavior (OHB) was positively (*r* = 0.14, *p* = 0.05) associated with OHK. There was a significant effect of education level (lower vs. higher) on OHK, (M^lower^ = 13.25 vs. M^higher^ = 13.99), F(1, 238) = 6.64, *p* = .011, SEd = 0.09, but not on OHB (*p* = 0.558). This not surprisingly, higher educated parents perceive more OHK from their children, but not more adequate OHB. Moreover, there was a significant effect of children age groups: 148 (61.7%) 5–7 years, 78 (32.5%) 8–10 years and 14 (5.8%) 11–12 years on the total OHB, (M^5–7year^ = 8.7 and M^8–10year^ = 8.6 vs. M^11–12year^ = 7.2), F(2, 237) = 3.74, *p* = .025, SEd = 1.97. In details there were only significant effects of children age groups on the frequency of toothbrushing, (M^5–7year^ = 1.6 and M^8–10year^ = 1.6 vs. M^11–12year^ = 1.1), F(2, 237) = 5.19, *p* = .006, SEd = 0.04, and on the time of day when the teeth are brushed, (M^5–7year^ = 2.1 and M^8–10year^ = 2.1 vs. M^11–12year^ = 1.4), F(2, 237) = 3.44, *p* = .034, SEd = 0.06. Children aged 11–12 years, referred to as adolescents; brush their teeth once a day, which is less frequently than children aged 5–10 years, who typically brush twice a day. Additionally, younger children tend to brush their teeth at the recommended times of day more consistently than those in the 11–12 age group. No differences were found within the age groups on OHK (*p* = 0.89).

To summarize, the gap between Oral Health Knowledge (OHK) and Oral Hygiene-Related Behavior (OHB) may be due to several factors, like parental education level and children's age. Although higher parental education is linked to better perceived OHK, it does not necessarily lead to more optimal OHB. Additionally, children's age plays a role, with older children (11–12 years) brushing less frequently and inconsistently than younger children, despite no difference in OHK. Parents may perceive their children's oral health knowledge positively, but this does not always translate into better brushing habits or behaviors, highlighting the need for behavioral interventions.

## Discussion

The aim of the study was to evaluate parents’ perspectives on their children's oral health knowledge (OHK) and oral hygiene behavior (OHB) among pre- and primary school children in Kaunas, Lithuania. The adapted OHB index showed that parents generally had good control over their children's brushing habits, with many brushing twice daily and using fluoride toothpaste as recommended worldwide (22). Also, parents demonstrated very good knowledge of their children's oral health care. The mean score on this adapted OHK index is comparable to the level of knowledge found in previous Dutch studies conducted among public people and military recruiters (12, 26), but much higher than found among an Indian population (27). This adapted OHB index for preschool and primary school children, based on optimal self-care recommendations by Lithuanian oral health experts (17), retains most elements of the original version (12), excluding one item on interdental cleaning, as this behavior is individualized and should be followed according to oral health professionals’ advice. Most participants were women, and they tended to use a manual toothbrush, reflecting a generally higher level of attention to oral health (28). The effectiveness of brushing, however, depends not only on the type of toothbrush—manual or powered—but also on how it is used. Here, it must be emphasized that the methods of using the powered brush under each technique are inappropriate and undesirable, and therefore are not recommended either. In addition, although vertical and circular toothbrushing methods were rated as less suitable by clinical experts (12, 13, 17), these methods were still common in the population studied. Many culturally adapted versions of the OHB index have been used in other contexts, and in the current study, the frequencies for the items in the OHB index for those who reported using a manual toothbrush (*n* = 240) closely match the reported oral hygiene behavior of a general Dutch population with a mean age of approximately 28 years in 2005 (12). Except on the items: ‘Frequency of toothbrushing’; in contrast to one third of the Lithuanian parents, 16% of the Dutch respondents reported brushing their teeth once a day and over 20% more reported brushing their teeth twice a day. On the item: ‘Pressure forces when brushing teeth’, more than twice as many Lithuanian respondents brushed their teeth more softly. Almost five times as many Dutch respondents brush according to the Bass method (12, 13), and half as many Lithuanian respondents brush their tongue daily. Ten years after the development of the original OHB index, in patients in York, Pennsylvania, USA in 2015 (23), a quarter of them brushed their teeth once daily, 7% brushed with force, 6% brushed their teeth with massaging movements near the gum line, and more than half of US respondents brushed their tongue at least once daily.

Optimal toothbrushing at a young age can lead to more effective oral hygiene habits in the future (13, 22, 29). It is known that poor oral hygiene may contribute to both local oral health issues, like cavities and gum disease, as well as systemic conditions such as cardiovascular disease and diabetes in old age. For instance, dysbiosis in the mouth's microbiota, especially interdental bacteria, can exacerbate these problems (30). Therefore, it is recommended to brush the child's teeth again until the age of 10. In this study, a third of parents consistently re-brushed their child's teeth after independent brushing, and parents exhibited strong knowledge of their children's oral health, as reflected in high scores on the OHK index. A positive correlation was found between OHB and OHK; parents with higher education demonstrated better OHK, but did not show improved OHB. This suggests that factors such as parental opinions and social health norms may have a greater influence on oral hygiene practices than knowledge alone. And interventions could also focus more on changing parental attitudes and promoting positive environmental factors that support good oral hygiene practices, and behavioral change to improve oral health outcomes and reduce global health risks (5, 29, 30).

The study has several limitations: it surveyed a group of parents in and around the city of Kaunas; both indices for OHB and OHK are modified in Lithuania, and its validity is uncertain. Moreover the data are based on reported behavior by parents, which may not always align with actual behavior of their children, and may be potentially influenced by socially desirable responses. Despite these limitations, this information is valuable for public health professionals, oral health professionals, and researchers aiming to improve children's oral health behavior. The modified oral hygiene-related behavior (OHB) index can be used to assess children's oral hygiene practices and parents’ knowledge across different populations. Recently, it was emphasized that the use of the original OHB index can be considered as one of the new approaches from the behavioral sciences that have the potential to change individual oral health behavior (31, 32). For public health professionals, the index helps identify specific needs for oral health education and tailor disease prevention programs. Oral health professionals can use it to assess and teach oral hygiene behavior and knowledge, identifying areas for improvement in individuals and to support them to change their behavior too.

In practice, this means that the Problem-Analysis-Test-Help-Success (PATHS) model should be applied correctly, with each phase playing a distinct role in designing effective interventions (33).

This means that in the Problem-phase, the potential target population should be defined. In the following Analysis- and Test-phase, a broad set of determinants of parental oral hygiene behavior and oral health knowledge for their young children should be assessed and analysed. Specifically, for the development of a tailored intervention in the Help phase, the findings from this study should focus on the population-specific determinants to inform the development of the intervention, such as a Smart Application designed to promote and help develop oral hygiene habits in children aged 5–12 years. And after all, in the Success-phase, the focus will be on evaluating the intervention to improve the Smart Application's effectiveness in real-world use, as well as for optimizing such a digital oral health intervention for promoting a desired oral self-care behavior in real-time, realworld settings (34). Based on the project's findings, a program will be developed to promote better oral hygiene and dietary habits in preschool and primary school children.

Education on oral health should follow an interdisciplinary approach (6), in line with WHO's vision of integrated health curricula for both oral health and allied health professionals. Public health concepts should be embedded in the training of all health professionals to enhance their understanding beyond clinical practice. Also, these findings could support (oral) health professionals working with different age groups in carrying out what are considered “the most dignified tasks” of their profession—educating these groups about oral health and promoting changes in their oral hygiene behavior. Dental hygienists, in particular, may play a key role in advancing oral hygiene behaviors and delivering preventive oral health messages on a global scale (9, 23, 29, 35). Further research done by dental hygienists, including longitudinal public oral health promotion studies in Lithuania, will be needed to refine and optimize these intervention strategies (36).

## Data Availability

The raw data supporting the conclusions of this article will be made available by the authors, without undue reservation.
